# Health seeking behaviours among construction workers: a systematic review

**DOI:** 10.1186/s12889-025-26173-8

**Published:** 2026-01-12

**Authors:** Saphiel Osei Poku, Kimblyn Anim, Biraso Poku Saphiel

**Affiliations:** 1 Department of Optometry and Visual Science, Faculty of Biosciences, College of Science, Kwame Nkrumah University of Science and Technology, Kumasi, Ghana; 2 Department of Urban Roads, Ashanti Region, Ministry of Roads and Highways, Kumasi, Ghana; 3 School of Nursing and Midwifery, Kumasi Campus, Wisconsin International University College, Kumasi, Ghana; 4 Department of History and Political Studies, Faculty of Social Sciences, College of Humanities and Social Sciences, Kwame Nkrumah University of Science and Technology, Kumasi, Ghana

**Keywords:** Health seeking behaviour, Healthcare utilization, Medical care seeking, Construction worker, Building worker

## Abstract

**Background:**

Construction workers face high levels of occupational health risks and hazards, yet little is known about their health seeking behaviours. Understanding the patterns, barriers, and facilitators of health seeking among these workers is essential to improve access to care and prevent adverse health outcomes. This systematic review explores the patterns of health seeking behaviours, the factors influencing such behaviours, assess impacts of the health seeking behaviours and effective interventions to improve health seeking among construction workers.

**Methods:**

We conducted a systematic review in accordance with the PRISMA 2020 guidelines. Eligible studies were identified through searches in PubMed, ScienceDirect, and Google Scholar from inception to January 14, 2025. Studies were selected via a PECOS framework, and the inclusion criteria comprised peer-reviewed quantitative and qualitative studies published in English. The risk of bias was assessed via appropriate JBI critical appraisal tools and the ROBINS-I tool. Data were extracted independently by two reviewers and synthesized thematically.

**Results:**

Of 1669 records identified, 16 studies met the inclusion criteria after screening and quality appraisal. Most studies were conducted in low- and middle-income countries with varied methodologies. Health seeking behaviours were classified into five predefined domains: reactive/illness-related, proactive/preventive, avoidance/delayed, informal, and mental health seeking. The predominant pattern was reactive health seeking following illness or injury. Barriers included lack of awareness, financial constraints, personal beliefs, limited workplace support, and healthcare inaccessibility. Facilitators included health education, workplace regulations, accessible health services and support systems. The impact of the health seeking behaviours included financial burdens, loss of worker days and psychosocial stress. Effective interventions included health and safety training, outreach programs, digital mental health tools, and improved occupational health policies.

**Conclusions:**

Construction workers predominantly engage in reactive health seeking, with significant barriers impeding timely and effective healthcare utilization. These behaviours result in preventable health and economic consequences. Interventions that improve awareness, accessibility, workplace policies, and mental health support are urgently needed. Future research should explore longitudinal patterns and evaluate context-specific interventions to improve health outcomes among construction workers.

**Trial registration:**

PROSPERO registration number CRD42025636549.

**Supplementary Information:**

The online version contains supplementary material available at 10.1186/s12889-025-26173-8.

## Background

Health is a complex concept that derives differing definitions across cultures, age groups and people with various life experiences [1]. It is beyond the scope of just being a lack of illness or disease and recognizes the interconnectedness among the physical, mental and social aspects of a person’s life and how they influence each other. As such, the World Health Organization defines health unanimously as a “state of complete physical, mental and social well-being and not just the mere absence of disease or infirmity” [2].

Health seeking behaviours are actions or inactions in the search of a remedy undertaken by individuals who perceive themselves to have a health problem [3]. Health seeking behaviours can also be defined as individual’s ability to promote maximum well-being, recovery and rehabilitation [4]. It is preceded by a decision-making process that is further governed by individual and/or household behaviour, community norms, and expectations, as well as provider-related characteristics and behaviour [3]. Utilization of the healthcare system and the process individuals follow in recognizing and responding to illness are the two approaches to health seeking behaviours [5]. Millions of people, especially children, die each year in low- and middle-income countries due to poor health seeking behaviours and delays in accessing biomedical treatment [6–8]. Inadequate financial resources, underresourced health care systems and delays in accessing medical care only partially explain the observed trends in the low uptake of optimal treatment patterns, which is a significant contributor to mortality and morbidity in low- and middle-income countries [9]. Individual health seeking behaviours are driven by vulnerability to poor health, healthcare expenses and the availability of resources, which have an economic impact on individual nations [10–14].

Construction workers form the second largest working group in the unorganized sector in India [15]. Construction works are physically demanding works performed in high risk environments, which can lead to a range of adverse effects [16, 17]. Cancers, back injuries, upper limb disorders and noise-related ill-health are some of the adverse effects affecting construction workers [18]. Construction worksites are termed as high risk environments because workers are exposed to many occupational health hazards, including harmful substances (i.e., clouds of dust, fumes, gases, and toxic chemicals), biological hazards (i.e., acute and chronic infections, and parasites), and physical hazards (i.e., noise, heat, cold, and vibration) [19]. The stressful and labor-intensive workplace and resource-limited conditions aggravate construction workers’ risk for poor health [20]. As such, the construction industry is one of the most hazardous sectors where health risks are significant [21]. Construction workers are prone to poor health, which worsens their economic conditions, often due to the deprivation of social security and employee benefits in view of their employment in the unorganized and informal sector [10].

There is significant evidence in the literature that the construction industry faces an increased prevalence of workplace stress, mental illness, suicidal thoughts and suicide attempts compared with other industry sectors [22]. This increased prevalence of health problems among construction workers underscores the need to synthesize existing evidence on their health seeking patterns and to examine how different health seeking behaviours may influence health outcomes. There is also a need to create awareness and enhance access to healthcare services, as inappropriate health seeking behaviours have been linked to worse health outcomes, increased morbidity and mortality and poorer health outcomes [23]. The prevalence rates of cancers, respiratory diseases, musculoskeletal disorders (MSDs) and noise-induced hearing loss (NIHL) are disproportionately high across the construction sectors of many countries in Europe and worldwide [24–32]. In the UK, the construction industry accounts for more than 40% of occupational cancer deaths and cancer registrations [18]. It has been estimated that past exposures in the construction sector annually cause over 5,000 occupational cancer cases and approximately 3,700 deaths in the UK [18] which can be mirrored worldwide. As such, this study identifies a need to improve the long-term health and quality of life of construction workers by identifying the barriers to and facilitators of health seeking behaviours among construction workers. This study also aims to gain more knowledge about effective safety regulations in the construction industry and to analyse the various effective interventions that can be adopted to improve health seeking behaviour among construction workers.

The main purpose of systematic review is to explore and synthesize the factors that influence access to and utilization of health seeking services and practices. It is generally understood that individuals’ perceptions of their health needs, the process of health decision-making and their concerns and considerations are key components in understanding health seeking behaviours [33]. Proper and extensive information is needed on existing health seeking behaviours to move towards higher quality healthcare structures [34].

The general objective of this study is to describe patterns of health seeking behaviours among construction workers. Specific objectives of the study includes: to identify barriers and facilitators influencing health seeking behaviours, to examine the impacts of health seeking behaviours, and to identify interventions aimed at improving it.

## Methods

### Protocol and registration

This systematic review followed the guidelines established by the Preferred Reporting Items for Systematic Reviews and Meta-analyses (PRISMA) [35, 36]. The systematic review was registered on 27 January 2025 with PROSPERO (CRD42025636549).

### Eligibility criteria

The PECOS framework guided the selection of literature for eligibility for this study. The PECOS acronym stands for population, exposure, comparator, outcome and study design. Only studies that met the criteria specified in PECOS were included. Five conceptual domains of health seeking behaviours were identified a priori from established theoretical and empirical literature: reactive/illness-related [37], proactive/preventive, avoidance/delayed [38], informal [39], and mental-health seeking [40]. These domains were used to guide the definition of exposures and outcomes during study selection, data extraction, and synthesis. Table 1 outlines the PECOS criteria used. We only included studies in English. Commentaries, reviews, protocols, editorial letters and grey literature sources (e.g., theses, reports, and non-peer-reviewed materials) were excluded to ensure the inclusion of studies that met peer-review and methodological quality standards.

**Table 1:** PECOS framework

Population	All age groups who are employed to work as construction workers
Exposure	Health seeking behaviours including proactive/preventive health seeking behaviours, reactive/illness-related health seeking behaviours, mental health seeking behaviours, avoidance or delayed health seeking behaviours and informal health seeking behaviours.
Comparator	Not applicable
Outcomes	General outcome: Patterns of health seeking behaviours among construction workers. Specific outcomes: Barriers and facilitators of health seeking behaviours among construction workers. Impact of poor health seeking behaviours among construction workers. Interventions to improve health seeking behaviours among construction workers. Effectiveness was assessed during full-text review and synthesis
Study design	Quantitative and Qualitative studies

### Search strategy

We conducted comprehensive searches in PubMed, Google Scholar and Science direct databases for all relevant studies from inception to January 14, 2025. We used the following search terms: ‘Health seeking behaviour’, ‘healthcare utilization’ and all applicable alternate terms crossed with ‘Construction worker’, ‘construction personnel’, ‘building worker’ and all applicable alternate terms. Boolean operators of “OR” and “AND” were used to increase the scope of studies eligible for screening. A detailed search strategy developed for all the databases used is listed in Supplementary Appendix 1.

### Selection process

The studies identified from the search were independently screened by two researchers via Rayyan Reference Manager. Each study had its title and abstract screened independently to obtain the relevant literature using the eligibility criteria. During the title and abstract screening, studies were included if they investigated, evaluated or proposed an intervention related to health seeking behaviours, without judging its effectiveness at this stage. Studies adhering to the eligibility criteria were full-text reviewed by two independent researchers and the reasons for exclusion at this stage were documented. Differences in the screening results for both stages were resolved by a third independent reviewer. For studies whose full texts were not accessible through open-access sources or institutional subscriptions, additional database and library searches were performed. 36 studies could not be retrieved: 14 were published between 1990 and 2014, for which journal links and author contact details were inactive, and 22 were recent papers (2019–2025) restricted by embargoes or subscription access. Direct author contact was not pursued for these papers due to the unavailability of valid contact information for older publications and the redundancy of topics covered by accessible studies. These 36 studies were therefore excluded and recorded in the PRISMA flow diagram.

### Quality assessment

We used the Joanna Briggs Institute (JBI) critical appraisal tools and the Risk of Bias in Non-randomized Studies of Interventions (ROBINS-I) tool to assess the risk of bias of the included full-text articles. The JBI checklist for analytical cross-sectional studies [41] is one of the JBI critical appraisal tools used, and it was used to assess the risk of bias of the included cross-sectional studies. We also used the JBI critical appraisal checklist for qualitative research [42] to assess the risk of bias of the included qualitative studies. The JBI critical appraisal checklist for studies reporting prevalence data [43] was also used to assess the risk of bias of the included prevalence study data. For the included case-control studies, we used the JBI critical appraisal checklist for case-control studies [41] to assess the risk of bias. The JBI critical appraisal checklist for cohort studies [41] was used to assess the risk of bias of the included cohort studies. The Risk of Bias in Non-randomized Studies of Interventions (ROBINS-I) tool was used to assess the risk of bias of the included intervention studies. Two independent reviewers assessed the risk of bias for each full-text article. Differences were resolved through a third independent reviewer. Studies rated as high risk of bias or failing to meet the minimum quality criteria were excluded at the synthesis stage. Specifically, eight studies with high overall risk of bias scores were omitted from the final analysis. The quality assessment checklist including the one for high risk of bias studies is presented in Supplementary Appendix 2–7.

### Data extraction and synthesis

Data were extracted independently by two reviewers from all eligible full-text studies. Extracted data were subsequently grouped and analyzed according to the five predefined domains of health seeking behaviours described above. Although the five domains of health seeking behaviours are conceptually interrelated, each included study was categorised under the single domain that best reflected its main research focus. This pragmatic approach facilitated clear synthesis while acknowledging potential conceptual overlap among domains. The data extracted included the first author, year of publication, study country, study design, sample size, sample population characteristics (age, gender), patterns of health seeking, barriers to health seeking, facilitators of health seeking, impacts of health seeking, effective interventions to promote good health seeking behaviours and the body system of health conditions studied are summarized in Table 2. Barriers and facilitators were identified across studies and grouped into conceptual themes (individual, interpersonal, workplace, and system-level factors). Impacts of health seeking behaviours and interventions to improve health seeking were reviewed thematically and categorized. A narrative thematic synthesis approach was applied to identify and integrate findings across studies.

**Table 2:** Summary of the characteristics of the included studies

Study ID	Author, Year	Study Designs	Study Country	Sample size	Population characteristics	
					Age	Gender (%)
Study 1	Wong (1994) [44]	Case control (Quantitative)	China	122	16–64	Males
Study 2	Robertson et al. (2007) [45]	Focus Groups (Qualitative)	USA	15	25–50	Males
Study 3	Dong et al. (2007) [46]	Observational (Quantitative)	USA	7025	16+	Males: 6358 (90.5%) Females: 667 (9.5%)
Study 4	Shuhaibar et al. (2011) [47]	Observational (Quantitative)	Ireland	909	53–60	Males
Study 5	Melo & Zago (2012) [48]	In-depth Interviews (Qualitative)	Brazil	8	18+	Males
Study 6	Naing et al. (2012) [49]	Cross-sectional (Quantitative)	Thailand	200	15–60	Males: 134 (62.6%) Females: 80 (37.4%)
Study 7	Tuma et al. (2013) [50]	Retrospective (Quantitative)	Qatar	281	17–76	Males
Study 8	Wang et al. (2014) [51]	Cross-sectional (Quantitative)	China	448	≤ 24–45	Males: 415 (92.6%) Females: 33 (7.4%)
Study 9	Shivalli et al. (2016) [52]	Cross-sectional (Quantitative)	India	119	18+	Males: 101 (85%) Females: 18 (15%)
Study 10	Getaneh (2016) [53]	Cross-sectional (Quantitative)	Ethiopia	650	18+	Males: 381 (58.6%) Females: 269 (41.4%)
Study 11	Yang et al. (2020) [54]	Retrospective (Quantitative)	USA	12,222	16+	Males: 11,714 (95.8%) Females: 508 (4.2%)
Study 12	Nwaogu et al. (2021) [55]	In-depth Interviews (Qualitative)	Nigeria	62	Mean age: 40	Males: 56 (90%) Females: 6 (10%)
Study 13	Boal et al. (2021) [56]	Cross-sectional (Quantitative)	USA	8567	18–64	N/A
Study 14	Aliyi et al. (2024) [57]	Cross-sectional (Quantitative)	Ethiopia	393	18–49	Males: 302 (76.8%) Females: 91 (23.2%)
Study 15	Darebo et al. (2024) [58]	Cross-sectional (Quantitative)	Ethiopia	412	15–49	Females
Study 16	Soundararajan et al. (2024) [59]	Cross-sectional (Quantitative)	India	1263	≥ 19	Males: 1145 (90.7%) Females: 102 (8.1%)

Studies with 100% male participants reflect the predominance of males in the construction sector and the absence of female workers in the study sample

## Results

**Fig. 1:**
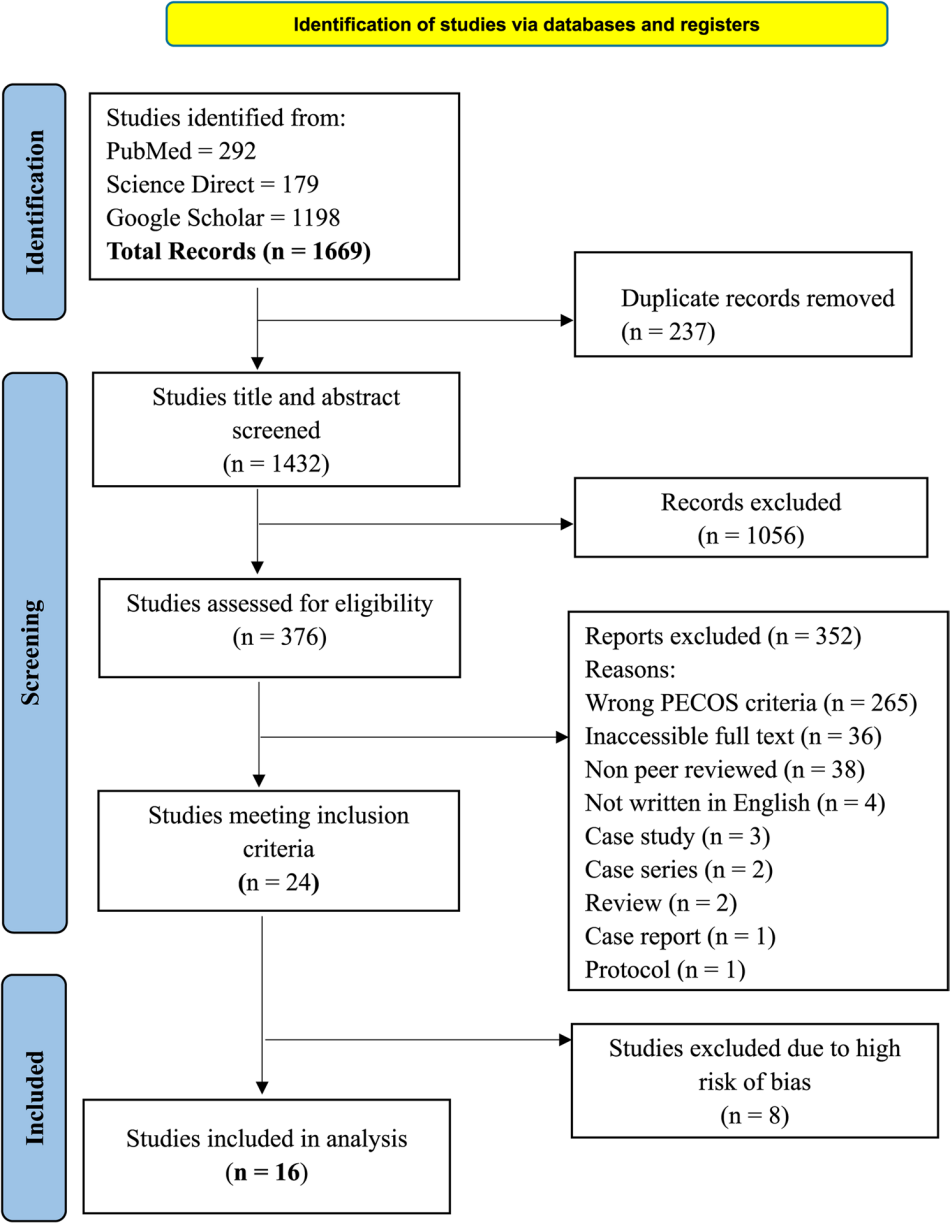
Flow diagram of study selection

The results are presented in a flow diagram according to the PRISMA guidelines (Fig. 1). The literature search revealed a total of 1669 studies. After removing 237 duplicate studies, 1432 studies remained for review on the basis of their titles and abstracts. Following this screening, 1056 studies were excluded, leaving 376 for eligibility assessment. Among these 376 studies, 352 studies were excluded. Among the 352 excluded studies, 265 did not meet our PECOS criteria as they did not address actual health seeking behaviours among construction workers, 36 studies whose full texts were inaccessible, 38 studies were not peer-reviewed, 4 studies were not written in English, 3 and 2 studies were case studies and case series respectively, 2 review studies were also excluded, and 1 protocol and 1 case report study were also excluded. 24 studies met the inclusion criteria, but 8 studies were excluded because of their high risk of bias. Common reasons for exclusion included unclear inclusion criteria, non-validated outcome measures, absence of confounder handling, unreported response rates, and reliance on convenience sampling. Detailed risk of bias profiles for these excluded studies is provided in Supplementary appendix 7. Ultimately, 16 studies were selected for the systematic review.

### Study characteristics

Sixteen studies published from 1994-2024 were included in this systematic review. These studies used diverse methodological designs, including quantitative study designs such as cross-sectional (*n* = 8), observational (*n* = 2), retrospective (*n* = 2) and case control (*n* = 1) designs. Additionally, 3 studies used qualitative study designs, including focus groups and in-depth interviews. The characteristics of the included studies are summarized in Table 2.

The included studies were conducted in various geographical locations, notably, the United States (*n* = 4), Ethiopia (*n* = 3), China (*n* = 2), India (*n* = 2) and others from Brazil, Ireland, Nigeria, Qatar, and Thailand. The populations studied consisted of male and female construction workers aged 15 + years, with sample sizes ranging from 8 to 12,222 participants. 

### Patterns of health seeking behaviours

Several studies examined how demographic and occupational characteristics shaped health seeking among construction workers. Across settings in low- and middle-income countries, workers most frequently preferred to seek care from government health facilities, which were viewed as more affordable and trustworthy than private clinics despite longer waiting times and variable service quality [52]. Gender differences were consistently reported: female construction workers, though under-represented in the overall workforce, demonstrated greater willingness to seek professional care particularly at public facilities whereas men more often delayed or relied on self-treatment [49]. Marital status also influenced health seeking: married workers reported more frequent use of healthcare services, attributing this to spousal encouragement and family responsibilities [49]. Age-related patterns were evident in that older workers (aged ≥ 20 years) accessed healthcare facilities more often than younger counterparts aged 15–19 years, who tended to normalise physical strain and perceive illness as a sign of weakness [58].

Health seeking behaviours among construction workers were grouped according to the five predefined domains. Each study was grouped under its predominant health seeking domain to maintain analytical clarity, while acknowledging that individual behaviours may span more than one domain. Table 3 depicts the health seeking behaviours of the included studies.

**Table 3:** Health seeking behaviours of the studies

Reactive/illness-related health seeking		
Study ID	Author, Year	Health condition, (Body system of condition)
Study 1	Wong (1994) [44]	Work-related injuries (Musculoskeletal system)
Study 3	Dong et al. (2007) [46]	Work-related injuries (Musculoskeletal system)
Study 5	Melo & Zago (2012) [48]	Silicosis (Respiratory system)
Study 7	Tuma et al. (2013) [50]	Work-related injuries (Musculoskeletal system)
Study 9	Shivalli et al. (2016) [52]	Malaria (circulatory system)
Study 11	Yang et al. (2020) [54]	Work-related injuries (Musculoskeletal system) Digestive diseases (Digestive system) Musculoskeletal diseases (Musculoskeletal system) Others
Study 13	Boal et al. (2021) [56]	N/A
Study 16	Soundararajan et al. (2024) [59]	N/A
Proactive/Preventive health seeking		
Study ID	Author, Year	Health condition, (Body system of condition)
Study 4	Shuhaibar et al. (2011) [47]	Colorectal cancer (Digestive system)
Study 10	Getaneh (2016) [53]	HIV/AIDS (Immune system)
Study 14	Aliyi et al. (2024) [57]	Work-related injuries (Musculoskeletal system)
Study 15	Darebo et al. (2024) [58]	Reproductive & Sexual health issues (Reproductive system)
Avoidance/Delayed health seeking		
Study ID	Author, Year	Health condition, (Body system of condition)
Study 2	Robertson et al. (2007) [45]	Hearing disorders (Auditory system)
Study 6	Naing et al. (2012) [49]	Obstetric & Gynecological issues (Reproductive system) Urinary tract issues (Excretory system) Respiratory issues (Respiratory system) Gastrointestinal issues (Digestive system) Others (General health issues)
Informal health seeking		
Study ID	Author, Year	Health condition, (Body system of condition)
Study 8	Wang et al. (2014) [51]	HIV/AIDS (Immune system)
Mental health seeking		
Study ID	Author, Year	Health condition, (Body system of condition)
Study 12	Nwaogu et al. (2021) [55]	Mental health issues (Nervous system)

Contextual details for each study (country, design, and population) are provided in Table 2

*Abbreviation*: *N/A* Not Applicable, *HIV/AIDS* Human Immunodeficiency Virus/Acquired Immunodeficiency Syndrome, *PPE* Personal Protective Equipment

Fifty percent (*n* = 8) of the included studies reported on Reactive/Illness-related health seeking behaviours [44, 46, 50, 52, 54, 56, 59, 60] where action was triggered only by the manifest onset of work-related injuries, respiratory conditions like silicosis, or other acute illnesses. Malaria, digestive diseases and musculoskeletal diseases also resulted in reactive health seeking. Reactive or illness-related health seeking behaviours were termed seeking care or remedy after the manifestation of a health problem or issue. Most studies reporting reactive behaviours originated from sub-Saharan Africa and South Asia, where construction work is largely informal and insurance coverage minimal. The predominance of male participants across these studies reflected the gender composition of the industry and reinforced cultural expectations of endurance and delayed health seeking. Quantitative cross-sectional designs dominated, providing consistent but context specific evidence that reactive care is the default response in informal construction settings.

This reactive stance appears to be a direct adaptation to the workers’ environment, overshadowing proactive/preventive behaviours [46, 52, 56, 57], which were less common and typically focused on mandatory or highly salient threats like HIV/AIDS or specific cancer screenings. Proactive/preventive health seeking behaviours accounted for 25% (*n* = 4) of the health seeking behaviours of the included studies [47, 53, 57, 58]. Proactive or preventive health seeking behaviours are defined as actions taken to maintain and improve health and to prevent diseases from occurring. The proactive/preventive health seeking behaviours included routine colorectal cancer screenings, personal protective equipment (PPE) and safety measures utilizations to safeguard against work-related injuries and HIV/AIDS, respectively. Health checks, vaccination, or participation in screening programmes occurred mainly in contexts where employers organised them on-site. Individual initiated prevention was rare and often limited to self-medication. Overall, preventive health seeking in construction remains incidental and externally driven, suggesting that structured workplace programmes are essential to normalise preventive care. The small number of studies, with modest sample sizes, suggests that proactive health seeking remains the exception rather than the norm globally.

The predominant reactivity was further compounded by patterns of avoidance and delay [45, 49]. Avoidance/delayed health seeking behaviours were defined as postponing medical care even after experiencing symptoms, signs or medical conditions that warrant treatment. Avoidance/delayed health seeking was observed in studies where workers were reluctant to seek care for health issues such as obstetric and gynecological issues, urinary tract issues, respiratory issues and gastrointestinal issues or exhibited a reluctance to use hearing protection [45, 49]. Some construction workers postponed seeking care due to fear of income loss, job insecurity, or the belief that symptoms would resolve spontaneously. Some avoided formal healthcare due to distrust or previous negative experiences. Workers postponing care, often normalized their symptoms as an inevitable part of their work.

Informal health seeking behaviours refer to the actions individuals take to address health concerns outside of the formal healthcare system. Informal health seeking including reliance on co-workers’ advice, over-the-counter drugs, and traditional healers was widely documented. One study found that construction workers preferred traditional or non-medical remedies [51]. Avoidance and informal health seeking were particularly common in studies from sub-Saharan Africa and South Asia, where most construction employment is informal and access to formal medical services is limited.

The most under represented pattern was mental health seeking [55], which was scarcely addressed, pointing to a critical gap in both research and practice. Mental health seeking behaviours are defined as the actions individuals take to address their psychological, social and emotional well-being. One study highlighted the potential of digital mental health interventions [55].

These patterns are not mutually exclusive but are interconnected manifestations of a workforce operating under significant constraints, where health is often sacrificed until a crisis point is reached. It was not always possible to differentiate clearly between health seeking related to occupational versus non-occupational conditions, as most primary studies did not specify the cause of the health issue prompting care. However, most of the reported behaviours concerned work-related problems such as injuries, respiratory or musculoskeletal complaints, and exposure to occupational hazards. A smaller number of studies addressed general or mental health concerns that were not directly attributed to workplace exposure. This overlap suggests that construction workers’ health seeking is influenced by both occupational and non-occupational needs, which may follow different pathways depending on workplace claims processes and access to compensation schemes. 

### Barriers to and facilitators of health seeking behaviours

Multiple individual, occupational, and systemic barriers were identified. Individual-level barriers included low education levels, lack of knowledge and awareness and personal beliefs. Interpersonal barriers involved lack of support from partners or family, workplace social behaviour (“don’t wear if others don’t wear”). Workplace and organisational barriers centred on lack of safety training, lack of workplace safety protocol, unavailability of PPE, absence of strict workplace regulation, job security fears. System-level barriers such as financial barriers, inaccessibility of healthcare services, lack of health insurance, lack of personal healthcare provider and lack of health card further discouraged health seeking. Structural and economic disincentives such as the financial burden of care, including direct costs, lack of health insurance and fear of lost wages were the most pervasive barriers. This was compounded by time constraints from demanding and inflexible work schedules, making clinic visits impractical. Socio-cultural norms and beliefs included deeply ingrained personal and cultural beliefs significantly shaped health seeking decisions. The masculine identity prevalent in construction, where seeking help was perceived as a sign of weakness and invulnerability to disease was assumed in some studies. Together, these findings reveal that structural and cultural constraints intersect to normalise delayed or avoided health seeking among construction workers. The most common barriers included lack of knowledge and awareness of occupational health issues (*n* = 6) and personal beliefs (*n* = 6). Some workers believe that they are invulnerable to auditory diseases because they are accustomed to excessive noise and believe that men are strong and that, health seeking as a man is a sign of weakness. The other personal beliefs held included not seeking care as symptoms were not serious enough, stigmatization from people towards seeking care for AIDS and a refusal to seek care owing to an inefficiency of an intervention. Aside from a lack of knowledge and personal belief as barriers to health seeking, other barriers included financial barriers such as an inability to afford care, time constraints due to demanding work schedules, unavailability and discomfort with the use of PPEs, a lack of safety training and a lack of workplace safety protocols. The detailed barriers to seeking health are summarized in Table 4. Table 4 includes only studies that explicitly identified at least one barrier or facilitator related to health seeking behaviour.

**Table 4:** Barriers and facilitators of health seeking among construction workers

Study ID	Author, Year	Barriers of health seeking	Facilitators of health seeking
Study 1	Wong (1994) [44]	Lack of safety training Low education levels	N/A
Study 2	Robertson et al. (2007) [45]	Personal belief (Being invulnerable to disease due to being accustomed to excessive noise) Lack of knowledge and awareness Discomfort with the use of protective equipment Job security fears Workplace social behaviour (don’t wear if others don’t wear)	Workplace regulations Awareness and knowledge of protective equipments
Study 5	Melo & Zago (2012) [48]	Lack of knowledge and awareness Personal belief (Being invulnerable to diseases, men are strong and seeking health is a sign of weakness) Inaccessibility of healthcare services	N/A
Study 6	Naing et al. (2012) [49]	Lack of health card Financial barriers Personal beliefs (symptoms not being serious) High levels of education (education levels above middle school)	Ease of access to health facilities
Study 8	Wang et al. (2014) [51]	Lack of knowledge and awareness Personal beliefs (negative attitudes towards AIDS patients)	N/A
Study 9	Shivalli et al. (2016) [52]	Lack of knowledge and awareness	N/A
Study 10	Getaneh (2016) [53]	Lack of workplace safety protocol Lack of safety training Unavailability of PPE Discomfort with the use of PPE Lack of support from partners	N/A
Study 12	Nwaogu et al. (2021) [55]	Lack of knowledge and awareness Financial barriers Personal belief (Inefficiency of intervention) Complicated usability Time constraints Intervention is boring due to lack of human interface	Efficiency & Effectiveness of intervention Motivational features of intervention
Study 13	Boal et al. (2021) [56]	Lack of health insurance Financial barriers Lack of personal healthcare provider	N/A
Study 14	Aliyi et al. (2024) [57]	Lack of knowledge and awareness Unavailability of PPE Discomfort with the use of protective equipment Absence of strict workplace regulation	N/A
Study 15	Darebo et al. (2024) [58]	N/A	Proximity to health facilities
Study 16	Soundararajan et al. (2024) [59]	Time constraints	N/A

*Abbreviations*: *HIV/AIDS* Human Immunodeficiency Virus/Acquired Immunodeficiency Syndrome, *N/A* Not Applicable, *PPE* Personal Protective Equipment

Facilitators identified in the studies similarly spanned several levels. Individual facilitators included awareness and knowledge of protective equipment, motivation features of interventions (reminders, feedback, or gamification elements that promote engagement), perceived efficiency and effectiveness of intervention (belief that the intervention works). No interpersonal facilitator was identified. Workplace-level facilitators involved enforcement and establishment of appropriate workplace regulations (e.g., enforcement of PPE use, safety policies). System-level facilitators encompassed ease of access to health facilities and proximity to health facilities. A key facilitator was the proximity and ease of access to healthcare services, which could overcome the structural hurdles. Facilitators that supported positive health seeking behaviours included awareness and knowledge of protective measures, supportive workplace policies, accessible health facilities, and well-designed educational or motivational interventions.

Synthesizing across studies suggests that individual motivation alone is insufficient to improve health seeking behaviours unless enabling organisational and system conditions are present. Programmes that combine worker education, peer support, and management commitment with improved service access show the greatest potential to overcome these barriers. This reinforces the need for integrated, multi-level interventions that address both behavioural and structural determinants.

Barriers and facilitators varied considerably across geographical and employment contexts. In low- and middle-income countries, financial constraints, absence of health insurance, and distance to facilities predominated as compared to high-income countries. The contextual contrasts illustrate how socioeconomic and organisational environments shape both obstacles and enablers of health seeking behaviour. Summary of barriers and facilitators across all levels are included in Tables 5 and 6.

**Table 5:** Barriers of health seeking behaviours across levels

Barriers of health seeking behaviours			
Individual barriers	Interpersonal barriers	Workplace barriers	System-level barriers
Low education levels Lack of knowledge and awareness Personal beliefs (being invulnerable to disease due to long exposure, men are strong and seeking health is a sign of weakness, inefficiency of of interventions, negative attitudes toward certain illnesses (e.g., HIV/AIDS, stigma) Discomfort with the use of protective equipment Complicated usability (difficulty using interventions or PPE) Time constraints (prioritising work over care) High levels of education (overconfidence or perceived lower susceptibility) Discomfort with the use of PPE	Lack of support from partners or family Workplace social behaviour (“don’t wear if others don’t wear”)	Lack of safety training Lack of workplace safety protocol Unavailability of PPE Absence of strict workplace regulation Job security fears	Financial barriers Inaccessibility of healthcare services Lack of health insurance Lack of personal healthcare provider Lack of health card

**Table 6:** Facilitators of health seeking behaviours across levels

Facilitators of health seeking behaviours			
Individual facilitators	Interpersonal facilitators	Workplace facilitators	System-level facilitators
Awareness and knowledge of protective equipment Motivation features of interventions (reminders, feedback, or gamification elements that promote engagement) Perceived efficiency and effectiveness of intervention (belief that the intervention works)		Workplace regulations (e.g., enforcement of PPE use, safety policies)	Ease of access to health facilities Proximity to health facilities

### Impact of health seeking behaviours

High financial burden and loss of worker days were the most identified impacts of health seeking behaviours. The total medical cost for construction workers was 1.36 billion US 2002 dollars in USA [46]. 4.4 million dollars in total were spent by construction workers on health and that each construction worker paid 15,735 dollars [50]. The psychosocial impacts of health seeking behaviours were recorded in the form of emotional distress and altered social identity [48]. Only [47] reported a positive impact of health seeking behaviours, whereby health seeking resulted in early detection of a disease (colorectal cancer).

Impacts of health seeking behaviours clustered across two inter-related domains. Psychological impacts included emotional distress from stress, anxiety, and diminished self-esteem associated with untreated conditions to increased confidence and perceived control among workers who accessed professional help. Economic impacts encompassed productivity loss due to loss of worker days but also cost savings and sustained employability when preventive or early interventions were used. Work loss following occupational injury or illness was reported in several studies. While such absences may reflect necessary recovery periods after diagnosis and treatment, prolonged or repeated absences can have economic implications for both workers and employers.

Table 7 gives a detailed breakdown of the impact of health seeking behaviours among construction workers. Only studies with at least one impact of health seeking or intervention to improve health seeking were included in Table 7.

**Table 7:** Impact of health seeking and interventions to improve health seeking behaviours

Study ID	Author, Year	Impacts of health seeking	Interventions to improve health seeking
Study 1	Wong (1994) [44]	High financial burden on workers and employers (mean healthcare cost was HKS10,676.93 (£928.43) Loss of worker days (range of sick leave 0–732 days)	Safety training programs
Study 3	Dong et al. (2007) [46]	High financial burden (total medical costs for all construction industry workers was $1.364 billion (2002 dollars) Loss of worker days	Improved worker compensation programs
Study 4	Shuhaibar et al. (2011) [47]	Early detection of disease (colorectal cancer)	Provision of national and pan European screening program (a standard two step approach using Fecal Immunochemical Test (FIT) for both screening)
Study 5	Melo & Zago (2012) [48]	Emotional distress Altered social identity such as voice change (perceived change in self-image and social role following illness or injury)	Improved access to health services (need to investigate risk group by occupational health nurses)
Study 6	Naing et al. (2012) [49]	N/A	Better TB screenings (to focus on active case finding not passive case finding) Improved healthcare education
Study 7	Tuma et al. (2013) [50]	High financial burden (Mean cost of care per admitted patient was approximately 16,000 USD)	Safety training programs (Fall prevention programs) Strict workplace regulation (about use of PPEs)
Study 8	Wang et al. (2014) [51]	N/A	Provision of healthcare facilities Improved health services (provision of treatment services) Increased educational programs Strengthening screening programs
Study 9	Shivalli et al. (2016) [52]	N/A	Increased educational programs
Study 10	Getaneh (2016) [53]	N/A	Provision of education and training
Study 11	Yang et al. (2020) [54]	High financial burden (average compensated medical cost was approximately $12000) Loss of worker days (ranges from 0–1696 days)	N/A
Study 12	Nwaogu et al. (2021) [55]	N/A	Digital mental health interventions
Study 14	Aliyi et al. (2024) [57]	Loss of worker days	Regular supervision and enforcement of PPE protocols
Study 15	Darebo et al. (2024) [58]	N/A	Outreach programs tailored to vulnerable groups
Study 16	Soundararajan et al. (2024) [59]	High financial burden	N/A

*Abbreviation*: *FIT* Fecal Immunochemical Test, *PPE* Personal Protective Equipment, *TB* Tuberculosis, *N/A* Not Applicable

### Interventions to improve health seeking behaviours

Interventions aimed at improving health seeking behaviours among construction workers fell broadly into two thematic categories. Educational and behavioural change programmes including safety and health promotion training were the most common. These interventions increased knowledge, awareness of risk, and proper use of personal protective equipment. Workplace-based and organisational strategies, such as regular supervision and enforcement of PPE protocols, provision of healthcare facilities such as on-site medical screening, mobile-clinic services, and employer-supported insurance or compensation schemes, improved access and early health seeking.

Taken together, the synthesis indicates that multicomponent approaches combining education, management commitment, and accessible health services produce the most consistent improvements in health seeking. Educational and safety training programs included targeted health literacy and fall prevention training [44, 49–53]. Outreach programs should be tailored to vulnerable or underserved groups for the early detection of high burden conditions [47, 49, 58]. Digital mental health tools have been identified as emerging intervention strategies to improve health seeking behaviours among construction workers [55]. Other recommended strategies include improved access to healthcare, worker compensation programs and enhanced workplace safety regulations [46, 48, 50, 51, 57]. Interventions were considered effective where the primary study authors reported positive outcomes such as improved health seeking behaviour, increased screening uptake, or enhanced adherence to safety protocols. Table 7 includes the effective interventions to improve the health seeking behaviours recommended by each study. Most evaluated interventions originated from high-income contexts. These included safety training and peer-support programmes with structured organisational backing. In low- and middle-income countries, evidence was mainly descriptive, recommending outreach or mobile-clinic initiatives rather than formally tested interventions. The concentration of empirical evaluation in well-resourced settings limits generalisability to informal and resource-constrained construction sectors.

## Discussion

This systematic review explored the patterns, barriers, facilitators, impacts and effective interventions related to health seeking behaviours among construction workers. There was a predominance of reactive or illness-related health seeking behaviours where workers sought treatment after the onset of a symptom, disease or injury [44, 46, 50, 52, 54, 56, 59, 60]. Preventive or proactive behaviours, including routine screenings and the use of protective equipment, are less common [47, 53, 57, 58]. This pattern suggests a critical gap in early intervention and disease prevention practices among construction workers. Avoidance or delayed health seeking behaviours are also observed, and are often influenced by personal beliefs, low perception of illness severity, time constraints, and stigma [45, 49]. Some workers believe that health seeking is a sign of weakness, especially among men, and that certain symptoms do not warrant medical attention [48]. Informal health seeking, such as relying on traditional medicine or non-professional advice, was present in some studies [51], highlighting mistrust in the formal healthcare system. Mental health seeking behaviour was the least represented in the reviewed literature, likely reflecting a research gap and the limited number of studies exploring mental health issues in the construction sector, rather than a definitive indication of low usage among workers.

The findings of this review are consistent with previous reviews on health seeking behaviours among the general working population, which similarly report low utilisation of preventive care [23]. Barriers such as job insecurity and limited access to occupational health services are well documented challenges in the construction sector [61]. Together, these factors appear to reinforce reactive rather than preventive patterns of health seeking.

The socio-demographic variations observed in this review underline the importance of tailoring health seeking interventions to the diversity within the construction workforce. The consistent finding that women were more likely than men to seek formal healthcare, particularly through public facilities, supports broader evidence that masculine norms of self-reliance discourage help seeking among male workers. Older and married workers also demonstrated greater health service utilisation, suggesting that family responsibilities and accumulated experience heighten perceived vulnerability and encourage treatment seeking. Conversely, younger and single workers showed stronger tendencies toward self-treatment or delayed care. This pattern likely reflects a combination of factors identified in this review, including lower perceived illness severity, financial constraints, job insecurity, time pressures, and socio-cultural norms that normalise endurance and discourage help-seeking. There should be interventions that account for gender and life-stage dynamics for example, integrating family or peer-support components and framing preventive care as compatible with productivity and resilience. Addressing these socio-demographic differences is therefore central to promoting equitable and sustained improvements in health seeking behaviour within the construction sector.

Key barriers to health seeking behaviours among construction workers include a lack of awareness, low education levels, and cultural beliefs [44, 45, 48, 51]. However [49], noted that high levels of education serve as a barrier to health seeking, as workers who have furthered their education beyond middle school tend to seek health less than people whose education level does not exceed middle school. Occupational barriers included poor safety training, lack of personal protective equipment (PPE), absence of workplace health policies, and fear of job insecurity [53, 57]. Other barriers, such as financial limitations, inaccessible health facilities and inadequate health insurance coverage, also contributed significantly [55, 56].

Facilitators of health seeking behaviours include increased awareness of occupational risks, proximity and access to healthcare services, supportive workplace regulations, and health education initiatives [45, 58]. When these enablers were present, improved health seeking behaviours were observed, emphasizing their importance in occupational health strategies.

The impact of health seeking behaviours was substantial. Most studies reported negative outcomes, such as high financial burdens, significant loss of productive workdays and psychological effects, including emotional distress and altered identity [46, 48, 50]. Only one study reported a positive outcome: early detection of colorectal cancer through a proactive screening program reinforcing the benefits of preventive care [47].

Evidence on effective interventions to improve health seeking among construction workers remains limited. The strongest sector specific evidence relates to the MATES in Construction peer-led programme, which has demonstrated improved help seeking attitudes and peer support [62]. However, the effective interventions identified in this review included safety and health education programs, digital mental health platforms, improved worker compensation programs, enhanced PPE protocols, and outreach initiatives for vulnerable worker groups [44, 50–53, 55, 58]. Reviews of occupational health and safety training, and workplace health promotion interventions in broader industries indicate that education and safety training can change safety practices and health behaviours, although training alone rarely yields large clinical improvements unless combined with management commitment, policy enforcement, and improved service access [63, 64]. These interventions reflect the necessity for an integrated, multifactorial approach to support the health of construction workers.

Overall, this review highlights the urgent need to address the unique health vulnerabilities of construction workers through inclusive policies, targeted interventions, and the promotion of a culture that supports timely and appropriate health seeking behaviours.

### Implications for research and practice

The findings of this review have important implications for both practice and future research. For practice, occupational health policymakers, employers, and construction sector stakeholders should prioritise multicomponent interventions that integrate safety training, peer support networks, access to on-site or mobile health services, and consistent enforcement of personal protective equipment use. Such approaches can help shift the prevailing reactive health seeking culture toward a more preventive one. For research, longitudinal and intervention studies are needed to assess the effectiveness of these strategies on actual health seeking behaviours, health outcomes, and productivity. Further investigation should also examine contextual factors such as employment stability, gender, and migration status that shape workers’ decisions to seek or delay care. The studies spanned multiple countries with most being from low- and middle-income settings, limiting their generalizability to high-income countries. Future research should include more studies from high-income countries to improve generalisability and support cross-contextual comparisons. Future studies should also prioritize addressing mental health seeking behaviours due to the limited evidence on mental health seeking behaviours despite the recognized burden of psychological distress in this sector.

### Limitations

This review has several limitations. First, only studies published in English were included, which may have introduced language bias. Second, gray literature, unpublished studies and non-peer-reviewed studies were excluded, potentially omitting relevant findings. Third, the included studies varied in design, methodology, and definitions of health seeking behaviours, which limit the comparability and synthesis of findings. Also, the review did not involve contacting study authors for inaccessible full texts. 14 of these were older papers with inactive access links, while 22 were recent publications restricted by embargo or paywall. Although this may have limited the comprehensiveness of the evidence base, the included studies adequately represented the range of health seeking behaviours among construction workers. Another important limitation is that few studies distinguished between occupational and non-occupational health problems, although the presence of compensation or claims mechanisms could substantially influence whether workers seek formal care. Future research should therefore analyse health seeking separately for occupational injuries and general health concerns to clarify how workplace systems shape these behaviours. Finally, although rigorous methods were applied, data extraction and synthesis may be subject to reviewer bias.

## Conclusion

This systematic review provides valuable insights into the health seeking behaviours of construction workers. These findings indicate that reactive behaviours are more common than preventive behaviours are and that multiple barriers hinder timely and effective health seeking. Cultural, occupational, individual and systemic factors collectively influence these behaviours. The health and economic consequences of health seeking especially poor health seeking are considerable, necessitating comprehensive and context-sensitive interventions. Future research should focus on mental health, longitudinal patterns of health seeking, and the effectiveness of tailored interventions to improve access to and utilization of healthcare services among construction workers.

## Electronic Supplementary Material

Below is the link to the electronic supplementary material.

Supplementary Material 1: Supplementary Appendix 1: Search strategies employed in this systematic review. Supplementary Appendix 2: Summary of risk of bias (JBI cross-sectional checklist). Supplementary Appendix 3: Summary of risk of bias (JBI Qualitative research checklist). Supplementary Appendix 4: Summary of risk of bias (JBI case-control research checklist). Supplementary Appendix 5: Summary of risk of bias (JBI Prevalence studies checklist). Supplementary Appendix 6: Summary of risk of bias (JBI Cohort studies checklist). Supplementary Appendix 7: Summary of risk of bias of high risk studies. (1) ROBINS-I Risk of Bias Assessment for King et al. (2018) [65]. (2) JBI Checklist for Analytical Cross-Sectional Studies for Stulhofer et al. (2006) [66]. (3) JBI Checklist for Analytical Cross-Sectional Studies for Patel et al. (2012) [67]. (4) JBI Checklist for Analytical Cross-Sectional Studies for Valsangkar et al. (2012) [68]. (5) JBI Checklist for Analytical Cross-Sectional Studies for Pracheth (2018) [69]. (6) JBI Checklist for Analytical Cross-Sectional Studies for Akram (2014) [70]. (7) JBI Prevalence studies checklist for Berríos-Torres et al. (2003) [71]. (8) JBI Prevalence studies checklist for Utuk and Atulomah (2023) [72].

## Data Availability

The datasets used and/or analysed during the current study are available from the corresponding author upon reasonable request.

## Abbreviations

MSD: Musculoskeletal disorder
NIHL: Noise-induced hearing loss
PRISMA: Preferred Reporting Items for Systematic Reviews and Meta-analyses
PECOS: Population, Exposure, Comparator, Outcome and Study design
JBI: Joanna Briggs Institute
ROBINS-I: Risk of Bias in Non-randomized Studies of Interventions
PPE: Personal Protective Equipment
