# Supplementary material for: Health seeking behaviours among construction workers: a systematic review
Source: BMC Public Health. 2026 Jan 12;26:520. doi: 10.1186/s12889-025-26173-8 (PMC12888678; doi:10.1186/s12889-025-26173-8)
Supplement: Supplementary file 1 — Supplementary Material 1: Supplementary Appendix 1: Search strategies employed in this systematic review. Supplementary Appendix 2: Summary of risk of bias (JBI cross-sectional checklist). Supplementary Appendix 3: Summary of risk of bias (JBI Qualitative research checklist). Supplementary Appendix 4: Summary of risk of bias (JBI case-control research checklist). Supplementary Appendix 5: Summary of risk of bias (JBI Prevalence studies checklist). Supplementary Appendix 6: Summary of risk of bias (JBI Cohort studies checklist). Supplementary Appendix 7: Summary of risk of bias of high risk studies. (1) ROBINS-I Risk of Bias Assessment for King et al. (2018) [65]. (2) JBI Checklist for Analytical Cross-Sectional Studies for Stulhofer et al. (2006) [66]. (3) JBI Checklist for Analytical Cross-Sectional Studies for Patel et al. (2012) [67]. (4) JBI Checklist for Analytical Cross-Sectional Studies for Valsangkar et al. (2012) [68]. (5) JBI Checklist for Analytical Cross-Sectional Studies for Pracheth (2018) [69]. (6) JBI Checklist for Analytical Cross-Sectional Studies for Akram (2014) [70]. (7) JBI Prevalence studies checklist for Berríos-Torres et al. (2003) [71]. (8) JBI Prevalence studies checklist for Utuk and Atulomah (2023) [72]. [file 12889_2025_26173_MOESM1_ESM.docx]

**Supplementary Appendix 1: Search strategies employed in this systematic review**

**Search in Science Direct:** search done multiple times with different search terms because of the limited number of words per search in the search engine.

| 1 | ("health seeking behavior" OR "health seeking behaviour" OR "health-seeking behavior") AND ("construction workers" OR "building workers" OR "construction laborers") | n = 23 |
| --- | --- | --- |
| 2 | ("health seeking behavior" OR "health seeking behaviour" OR "health-seeking behavior") AND ("construction labourers" OR "construction workforce" OR "construction personnel") | n = 1 |
| 3 | ("health-seeking behaviour" OR "healthcare utilization" OR "health service utilization") AND ("construction workers" OR "building workers" OR "construction laborers") | n = 53 |
| 4 | ("health-seeking behaviour" OR "healthcare utilization" OR "health service utilization") AND ("construction labourers" OR "construction workforce" OR "construction personnel") | n = 1 |
| 5 | ("medical-care seeking" OR "medical care seeking" OR "medical help seeking") AND ("construction workers" OR "building workers" OR "construction laborers") | n = 2 |
| 6 | ("medical-care seeking" OR "medical care seeking" OR "medical help seeking") AND ("construction labourers" OR "construction workforce" OR "construction personnel") | n = 0 |
| 7 | ("medical-help seeking" OR "healthcare access" OR "health service access") AND ("construction workers" OR "building workers" OR "construction laborers") | n = 27 |
| 8 | ("medical-help seeking" OR "healthcare access" OR "health service access") AND ("construction labourers" OR "construction workforce" OR "construction personnel") | n = 2 |
| 9 | ("help seeking" OR "help-seeking") AND ("construction workers" OR "building workers" OR "construction laborers") | n = 57 |
| 10 | ("help seeking" OR "help-seeking") AND ("construction labourers" OR "construction workforce" OR "construction personnel") | n = 13 |
|  | Total studies | n = 179 |

**Search in PubMed:**

| #1 | (("health"[MeSH Terms] OR "health"[All Fields] OR "health s"[All Fields] OR "healthful"[All Fields] OR "healthfulness"[All Fields] OR "healths"[All Fields]) AND ("seeking"[All Fields] OR "seeks"[All Fields]) AND ("behavior"[MeSH Terms] OR "behavior"[All Fields] OR "behavioral"[All Fields] OR "behavioural"[All Fields] OR "behavior s"[All Fields] OR "behaviorally"[All Fields] OR "behaviour"[All Fields] OR "behaviourally"[All Fields] OR "behaviours"[All Fields] OR "behaviors"[All Fields] OR "pattern"[All Fields] OR "pattern s"[All Fields] OR "patternability"[All Fields] OR "patternable"[All Fields] OR "patterned"[All Fields] OR "patterning"[All Fields] OR "patternings"[All Fields] OR "patterns"[All Fields])) OR (("health"[MeSH Terms] OR "health"[All Fields] OR "health s"[All Fields] OR "healthful"[All Fields] OR "healthfulness"[All Fields] OR "healths"[All Fields]) AND ("seeking"[All Fields] OR "seeks"[All Fields]) AND ("behavior"[MeSH Terms] OR "behavior"[All Fields] OR "behavioral"[All Fields] OR "behavioural"[All Fields] OR "behavior s"[All Fields] OR "behaviorally"[All Fields] OR "behaviour"[All Fields] OR "behaviourally"[All Fields] OR "behaviours"[All Fields] OR "behaviors"[All Fields] OR "pattern"[All Fields] OR "pattern s"[All Fields] OR "patternability"[All Fields] OR "patternable"[All Fields] OR "patterned"[All Fields] OR "patterning"[All Fields] OR "patternings"[All Fields] OR "patterns"[All Fields])) OR ("health-seeking"[All Fields] AND ("behavior"[MeSH Terms] OR "behavior"[All Fields] OR "behavioral"[All Fields] OR "behavioural"[All Fields] OR "behavior s"[All Fields] OR "behaviorally"[All Fields] OR "behaviour"[All Fields] OR "behaviourally"[All Fields] OR "behaviours"[All Fields] OR "behaviors"[All Fields] OR "pattern"[All Fields] OR "pattern s"[All Fields] OR "patternability"[All Fields] OR "patternable"[All Fields] OR "patterned"[All Fields] OR "patterning"[All Fields] OR "patternings"[All Fields] OR "patterns"[All Fields])) OR ("health-seeking"[All Fields] AND ("behavior"[MeSH Terms] OR "behavior"[All Fields] OR "behavioral"[All Fields] OR "behavioural"[All Fields] OR "behavior s"[All Fields] OR "behaviorally"[All Fields] OR "behaviour"[All Fields] OR "behaviourally"[All Fields] OR "behaviours"[All Fields] OR "behaviors"[All Fields] OR "pattern"[All Fields] OR "pattern s"[All Fields] OR "patternability"[All Fields] OR "patternable"[All Fields] OR "patterned"[All Fields] OR "patterning"[All Fields] OR "patternings"[All Fields] OR "patterns"[All Fields])) OR (("delivery of health care"[MeSH Terms] OR ("delivery"[All Fields] AND "health"[All Fields] AND "care"[All Fields]) OR "delivery of health care"[All Fields] OR "healthcare"[All Fields] OR "healthcare s"[All Fields] OR "healthcares"[All Fields]) AND ("statistics and numerical data"[MeSH Subheading] OR ("statistics"[All Fields] AND "numerical"[All Fields] AND "data"[All Fields]) OR "statistics and numerical data"[All Fields] OR "utilization"[All Fields] OR "utilisation"[All Fields] OR "utilisations"[All Fields] OR "utilise"[All Fields] OR "utilised"[All Fields] OR "utilises"[All Fields] OR "utilising"[All Fields] OR "utilities"[All Fields] OR "utility"[All Fields] OR "utilizations"[All Fields] OR "utilize"[All Fields] OR "utilized"[All Fields] OR "utilizer"[All Fields] OR "utilizers"[All Fields] OR "utilizes"[All Fields] OR "utilizing"[All Fields])) OR (("health services"[MeSH Terms] OR ("health"[All Fields] AND "services"[All Fields]) OR "health services"[All Fields] OR ("health"[All Fields] AND "service"[All Fields]) OR "health service"[All Fields]) AND ("statistics and numerical data"[MeSH Subheading] OR ("statistics"[All Fields] AND "numerical"[All Fields] AND "data"[All Fields]) OR "statistics and numerical data"[All Fields] OR "utilization"[All Fields] OR "utilisation"[All Fields] OR "utilisations"[All Fields] OR "utilise"[All Fields] OR "utilised"[All Fields] OR "utilises"[All Fields] OR "utilising"[All Fields] OR "utilities"[All Fields] OR "utility"[All Fields] OR "utilizations"[All Fields] OR "utilize"[All Fields] OR "utilized"[All Fields] OR "utilizer"[All Fields] OR "utilizers"[All Fields] OR "utilizes"[All Fields] OR "utilizing"[All Fields])) OR (("med care"[Journal] OR ("medical"[All Fields] AND "care"[All Fields]) OR "medical care"[All Fields]) AND ("seeking"[All Fields] OR "seeks"[All Fields])) OR (("med care"[Journal] OR ("medical"[All Fields] AND "care"[All Fields]) OR "medical care"[All Fields]) AND ("seeking"[All Fields] OR "seeks"[All Fields])) OR (("medic"[All Fields] OR "medical"[All Fields] OR "medicalization"[MeSH Terms] OR "medicalization"[All Fields] OR "medicalizations"[All Fields] OR "medicalize"[All Fields] OR "medicalized"[All Fields] OR "medicalizes"[All Fields] OR "medicalizing"[All Fields] OR "medically"[All Fields] OR "medicals"[All Fields] OR "medicated"[All Fields] OR "medication s"[All Fields] OR "medics"[All Fields] OR "pharmaceutical preparations"[MeSH Terms] OR ("pharmaceutical"[All Fields] AND "preparations"[All Fields]) OR "pharmaceutical preparations"[All Fields] OR "medication"[All Fields] OR "medications"[All Fields]) AND "help"[All Fields] AND ("seeking"[All Fields] OR "seeks"[All Fields])) OR ("medical-help"[All Fields] AND ("seeking"[All Fields] OR "seeks"[All Fields])) OR (("delivery of health care"[MeSH Terms] OR ("delivery"[All Fields] AND "health"[All Fields] AND "care"[All Fields]) OR "delivery of health care"[All Fields] OR "healthcare"[All Fields] OR "healthcare s"[All Fields] OR "healthcares"[All Fields]) AND ("access"[All Fields] OR "accessed"[All Fields] OR "accesses"[All Fields] OR "accessibilities"[All Fields] OR "accessibility"[All Fields] OR "accessible"[All Fields] OR "accessing"[All Fields])) OR (("health services"[MeSH Terms] OR ("health"[All Fields] AND "services"[All Fields]) OR "health services"[All Fields] OR ("health"[All Fields] AND "service"[All Fields]) OR "health service"[All Fields]) AND ("access"[All Fields] OR "accessed"[All Fields] OR "accesses"[All Fields] OR "accessibilities"[All Fields] OR "accessibility"[All Fields] OR "accessible"[All Fields] OR "accessing"[All Fields])) | n = 1,506,047 |
| --- | --- | --- |
| #2 | "construction workers"[All Fields] OR "building workers"[All Fields] OR "construction laborers"[All Fields] OR "construction workforce"[All Fields] OR "construction personnel"[All Fields] OR "construction labourers"[All Fields] | n = 2339 |
| #3 | Search: (#1) AND (#2) | n = 279 |
| #4 | ("help-seeking"[All Fields] OR "help-seeking"[All Fields]) AND ("construction workers"[All Fields] OR "building workers"[All Fields] OR "construction laborers"[All Fields] OR "construction labourers"[All Fields] OR "construction workforce"[All Fields] OR "construction personnel"[All Fields]) | n = 13 |
|  | Total studies | n = 292 |

**Search in Google scholar via Harzing’s Publish or Perish:**

| 1 | "health seeking behavior" OR "healthcare utilization" OR "health service utilization" OR "medical care seeking" OR "medical help seeking" OR "healthcare access" OR "health service access" "construction workers" | n = 998 |
| --- | --- | --- |
| 2 | "help seeking" OR "help-seeking" "construction workers" OR "building workers" OR "construction laborers" OR "construction labourers" OR "construction workforce" OR "construction personnel" | n = 200 |
|  | Total | n = 1198 |

**Supplementary Appendix 2: Summary of risk of bias (JBI cross-sectional checklist)**

| Risk of Bias | (46) | (49) | (51) | (52) | (53) | (54) | (56) | (57) | (58) | (59) |
| --- | --- | --- | --- | --- | --- | --- | --- | --- | --- | --- |
| Clearly  Defined  Inclusion  Criteria |  |  |  |  |  |  |  |  |  |  |
| Detailed  Description of  Study subjects  & Settings |  |  |  |  |  |  |  |  |  |  |
| Validity &  Reliability of  Measured  Exposure |  |  |  |  |  |  |  |  |  |  |
| Standard Objective  Measurement  Of Condition |  |  |  |  |  |  |  |  |  |  |
| Confounding  Factors  Identified |  |  |  |  |  |  |  |  |  |  |
| Confounding Strategies  Stated |  |  |  |  |  |  |  |  |  |  |
| Validity &  Reliability of  Measured  Outcomes |  |  |  |  |  |  |  |  |  |  |
| Appropriate  Statistical analysis |  |  |  |  |  |  |  |  |  |  |
| Overall  Appraisal | Include | Include | Include | Include | Include | Include | Include | Include | Include | Include |

| Yes | No | Unclear | Not applicable |
| --- | --- | --- | --- |

**Supplementary Appendix 3: Summary of risk of bias (JBI Qualitative research checklist)**

| Risk of Bias | (45) | (48) | (55) |
| --- | --- | --- | --- |
| Congruity between the stated philosophical perspective and research methodology |  |  |  |
| Congruity between the research methodology and the Research question or objectives |  |  |  |
| Congruity between the research methodology and data collection methods |  |  |  |
| Congruity between the research methodology and data representation and analysis |  |  |  |
| Congruity between the research methodology and the interpretation of results |  |  |  |
| Statement locating the researcher culturally or theoretically |  |  |  |
| Influence of the researcher on the research, and vice- versa, addressed |  |  |  |
| Adequate representation of participants and their voices |  |  |  |
| Ethicality of research according to current criteria, and is there evidence of ethical approval  by an appropriate body |  |  |  |
| Conclusions drawn in the research report flow from the analysis, or interpretation, of the data |  |  |  |
| Overall Appraisal | Include | Include | Include |

| Yes | No | Unclear | Not applicable |
| --- | --- | --- | --- |

**Supplementary Appendix 4: Summary of risk of bias (JBI case-control research checklist)**

| Risk of Bias | (44) |
| --- | --- |
| Were the groups comparable other than the presence of disease in cases or the absence of disease in controls? |  |
| Were cases and controls matched appropriately? |  |
| Were the same criteria used for identification of cases and controls? |  |
| Was exposure measured in a standard, valid and reliable way? |  |
| Was exposure measured in the same way for cases and controls? |  |
| Were confounding factors identified? |  |
| Were strategies to deal with confounding factors stated? |  |
| Were outcomes assessed in a standard, valid and reliable way for cases and controls? |  |
| Was the exposure period of interest long enough to be meaningful? |  |
| Was appropriate statistical analysis used? |  |
| Overall Appraisal | Include |

| Yes | No | Unclear | Not applicable |
| --- | --- | --- | --- |

**Supplementary Appendix 5: Summary of risk of bias (JBI Prevalence studies checklist)**

| Risk of Bias | (50) |
| --- | --- |
| Was the sample frame appropriate to address the target population? |  |
| Were study participants sampled in an appropriate way? |  |
| Was the sample size adequate? |  |
| Were the study subjects and the setting described in detail? |  |
| Was the data analysis conducted with sufficient coverage of the identified sample? |  |
| Were valid methods used for the identification of the condition? |  |
| Was the condition measured in a standard, reliable way for all participants? |  |
| Was there appropriate statistical analysis? |  |
| Was the response rate adequate, and if not, was the low response rate managed appropriately? |  |
| Overall Appraisal | Include |

| Yes | No | Unclear | Not applicable |
| --- | --- | --- | --- |

**Supplementary Appendix 6: Summary of risk of bias (JBI Cohort studies checklist)**

| Risk of Bias | (47) |
| --- | --- |
| Were the two groups similar and recruited from the same population? |  |
| Were the exposures measured similarly to assign people to both exposed and unexposed groups? |  |
| Was the exposure measured in a valid and reliable way? |  |
| Were confounding factors identified? |  |
| Were strategies to deal with confounding factors stated? |  |
| Were the groups/participants free of the outcome at the start of the study (or at the moment of exposure)? |  |
| Were the outcomes measured in a valid and reliable way? |  |
| Was the follow up time reported and sufficient to be long enough for outcomes to occur? |  |
| Was follow up complete, and if not, were the reasons to loss to follow up described and explored? |  |
| Were strategies to address incomplete follow up utilized? |  |
| Was appropriate statistical analysis used? |  |
| Overall Appraisal | Include |

| Yes | No | Unclear | Not applicable |
| --- | --- | --- | --- |

**Supplementary Appendix 7: Summary of risk of bias of high risk studies**

1. **ROBINS-I Risk of Bias Assessment for** **King et al. (2018) (65)**

| Domain | Risk of Bias | Justification |
| --- | --- | --- |
| 1. Bias due to confounding |  | No control group. Possible confounders  (e.g., prior beliefs, mental health history) were  not adjusted. Only age, sex, location, and training year were controlled. Can't rule out that other factors  caused the observed belief changes. |
| 2. Bias in selection of participants into the study |  | All workers at recruited sites were invited, but participation bias is likely (those more open to  mental health training may have been more  likely to attend). Excluded repeat attendees and  those with incomplete data. |
| 3. Bias in classification of interventions |  | All participants received the same intervention  (GAT), and classification is accurate. |
| 4. Bias due to deviations from  intended interventions |  | No blinding. Trainers may have varied in delivery, and implementation fidelity was not assessed.  Clustering was adjusted statistically but not  monitored in delivery. |
| 5. Bias due to missing data |  | 33% of the eligible sample was excluded  due to missing pre/post responses or  demographic info. Missingness may be  non-random (e.g., more among women and  in certain states). |
| 6. Bias in measurement of outcomes |  | Self-reported beliefs, measured immediately  post-intervention. High risk of social desirability  bias, no long-term follow-up, and no  blinding to outcomes. |
| 7. Bias in selection of the reported result |  | No registered protocol. Only 4 belief items  were assessed, and help-seeking intent was not measured pre-post. Risk of selective  reporting is present. |

| Yes | No | Unclear | Not applicable |
| --- | --- | --- | --- |

1. **JBI Checklist for Analytical Cross-Sectional Studies** **for Stulhofer et al. (2006) (66)**

| JBI Appraisal Criteria | Response | Justification |
| --- | --- | --- |
| Were the criteria for inclusion in the  sample clearly defined? |  | Eligibility criteria (migrant workers  undergoing mandatory medical examinations)  were described. |
| Were the study subjects and the  setting described in detail? |  | Participant demographics, occupations, and  recruitment sites (seven clinics across Croatia)  were detailed. |
| Was the exposure measured in a  valid and reliable way? |  | HIV-related knowledge and attitudes were  measured using a non-validated,  self-constructed questionnaire. |
| Were objective, standard criteria  used for measurement of the condition? |  | Risk behaviors and STI history were self-reported,  with no verification or standardized clinical data. |
| Were confounding factors identified? |  | Some potential confounders (age, education,  marital status, religiosity) were mentioned  and statistically controlled, residual  confounding remained substantial |
| Were strategies to deal with  confounding factors stated? |  | Regression models were used, but residual  confounding was acknowledged, not all  relevant variables were controlled. |
| Were the outcomes measured in a  valid and reliable way? |  | Outcomes such as condom use and number of  sexual partners were self-reported, subject to  recall and social desirability bias. |
| Was appropriate statistical analysis used? |  | Descriptive and logistic regression analyses were  applied appropriately using SPSS. |

| Yes | No | Unclear | Not applicable |
| --- | --- | --- | --- |

1. **JBI Checklist for Analytical Cross-Sectional Studies for Patel et al. (2012) (67)**

| JBI Appraisal Criteria | Response | Comments / Justification |
| --- | --- | --- |
| Were the criteria for inclusion in  the sample clearly defined? |  | The study did not describe specific inclusion or  exclusion criteria beyond being “male  construction workers” at selected sites. |
| Were the study subjects and the  setting described in detail? |  | Sociodemographic details, location (three sites in  Surat city), and work characteristics were reported. |
| Was the exposure measured in a  valid and reliable way? |  | Exposures such as “working conditions,” “habits,” and “type of work” were based on self-reports  without standardized instruments or validation. |
| Were objective, standard criteria  used for measurement of the  condition? |  | Health problems were self-reported; no clinical  verification or standardized criteria were applied. |
| Were confounding factors  identified? |  | The study did not explicitly identify potential  confounders such as age, education,  duration of employment, or socioeconomic status. |
| Were strategies to deal with  confounding factors stated? |  | No adjustment or stratified analysis was performed;  only bivariate chi-square tests were used. |
| Were the outcomes measured in a  valid and reliable way? |  | Outcomes (morbidity, habits, satisfaction) relied  on unvalidated self-report data, likely subject to recall  and social desirability bias. |
| Was appropriate statistical  analysis used? |  | Only descriptive and chi-square analyses were  used; regression or multivariable analyses were  absent. Statistical reporting was basic. |

| Yes | No | Unclear | Not applicable |
| --- | --- | --- | --- |

1. **JBI Checklist for Analytical Cross-Sectional Studies for Valsangkar et al. (2012) (68)**

| JBI Appraisal Criteria | Response | Comments / Justification |
| --- | --- | --- |
| Were the criteria for inclusion in  the sample clearly defined? |  | Inclusion criteria were stated: construction workers at  two sites who consented to participate, excluding those  with prior musculoskeletal injury or trauma. |
| Were the study subjects and the  setting described in detail? |  | Study described number of sites, job types,  gender distribution, and socioeconomic  classification (Kuppuswamy scale). |
| Was the exposure measured in a  valid and reliable way? |  | Musculoskeletal disorders defined using a  validated operational definition, but exposure  assessment relied on self-report without  clinical verification. |
| Were objective, standard criteria  used for measurement of the  condition? |  | Musculoskeletal disorders defined using validated  National Institute for Occupational Safety and Health  criteria. However, assessments were  self-reported without physical examination or  diagnostic confirmation. |
| Were confounding factors  identified? |  | Authors identified socioeconomic status, job  type, community support, housing, and  sanitation as potential social determinants  influencing health outcomes. |
| Were strategies to deal with  confounding factors stated? |  | Binary logistic regression was conducted including  social determinant domains as predictors, but  individual confounders were not adjusted  comprehensively. |
| Were the outcomes measured in a  valid and reliable way? |  | Outcomes (physical and mental health, activity  limitation) were assessed using the Centers for Disease  Control and Preventions’ “Healthy Days Module,”  which is validated, but implementation and  interviewer training were not detailed. |
| Was appropriate statistical  analysis used? |  | Descriptive statistics and logistic regression  were appropriately used. Reporting included  Nagelkerke R² value |

| Yes | No | Unclear | Not applicable |
| --- | --- | --- | --- |

1. **JBI Checklist for Analytical Cross-Sectional Studies for Pracheth (2018)** **(69)**

| JBI Appraisal Criteria | Response | Comments / Justification |
| --- | --- | --- |
| Were the criteria for inclusion in  the sample clearly defined? |  | Inclusion criteria (workers aged ≥18 years at  selected construction sites) were mentioned, but no  exclusion criteria or recruitment process details were  provided. |
| Were the study subjects and the  setting described in detail? |  | The study described participant demographics (age,  gender, education, income, duration in Mangalore) and  setting (urban construction sites in Mangalore). |
| Was the exposure measured in a  valid and reliable way? |  | Health and morbidity data were obtained through  self-reported interviews using a semi-structured  questionnaire without validation of medical conditions. |
| Were objective, standard criteria  used for measurement of the  condition? |  | No standardized diagnostic criteria or clinical  assessments were used to confirm reported illnesses or  injuries. |
| Were confounding factors  identified? |  | Potential confounders such as age, duration of  employment, income, or type of work were collected  but not identified or discussed as confounders. |
| Were strategies to deal with  confounding factors stated? |  | Only descriptive statistics were used; no  stratified or multivariate analyses were performed to  control for confounders. |
| Were the outcomes measured in a  valid and reliable way? |  | Outcomes like “fever,” “skin disease,” and “injury” were  self-reported retrospectively for one year, which is  prone to recall bias and misclassification. |
| Was appropriate statistical  analysis used? |  | Descriptive and simple percentage analyses were  used; appropriate for exploratory design but  inadequate for assessing associations or causality. |

| Yes | No | Unclear | Not applicable |
| --- | --- | --- | --- |

1. **JBI Checklist for Analytical Cross-Sectional Studies for** **Akram (2014) (70)**

| JBI Appraisal Criteria | Response | Comments / Justification |
| --- | --- | --- |
| Were the criteria for inclusion in  the sample clearly defined? |  | Inclusion/exclusion criteria not clearly stated, workers  simply “available” at sites. |
| Were the study subjects and the  setting described in detail? |  | Basic demographics and work characteristics reported. |
| Was the exposure measured in a  valid and reliable way? |  | Safe-practice exposure measured using unvalidated  self-constructed questionnaire. |
| Were objective, standard criteria  used for measurement of the  condition? |  | Knowledge and attitude cut-offs appear arbitrary, no  validated scoring system. |
| Were confounding factors  identified? |  | Potential confounders (age, training, experience,  PPE availability) not acknowledged. |
| Were strategies to deal with  confounding factors stated? |  | No statistical adjustment (regression/stratification)  performed. |
| Were the outcomes measured in a  valid and reliable way? |  | Questionnaire reliability not reported, no pre-test  reliability statistics. |
| Was appropriate statistical  analysis used? |  | Basic descriptive analysis appropriate, however no  inferential tests applied to explain relationships. |

| Yes | No | Unclear | Not applicable |
| --- | --- | --- | --- |

1. **JBI Prevalence studies checklist for** **Berríos-Torres et al. (2003) (71)**

| Risk of Bias | | Response | | Justification | | |
| --- | --- | --- | --- | --- | --- | --- |
| Was the sample frame appropriate to address  the target population? | |  | | Only workers formally assigned to rescue duties  included. Others with similar exposures excluded. | | |
| Were study participants sampled in  an appropriate way? | |  | | Convenience sampling based on clinical  presentation to specific facilities, not population-based. | | |
| Was the sample size adequate? | |  | | Large number of rescue worker visits captured  over surveillance period. | | |
| Were the study subjects and the setting  described in detail? | |  | | Worker types, demographics, facility distribution,  and encounter characteristics documented. | | |
| Was the data analysis conducted with  sufficient coverage of the identified sample? | |  | | Not all treatment locations included (ambulances, other  emergency departments, respite centers). 13% of  forms missing. | | |
| Were valid methods used for the  identification of the condition? | |  | | Clinician-recorded medical assessments at point of care. | | |
| Was the condition measured in a  standard, reliable way for all participants? | |  | | No standardized diagnostic criteria. Missing  PPE/activity variables (80–93%). | | |
| Was there appropriate statistical analysis? | |  | | Standard incidence rate calculations applied,  though denominators uncertain. | | |
| Was the response rate adequate, and if not,  was the low response rate  managed appropriately? | |  | | Missing data not formally addressed; incomplete  capture acknowledged but not corrected. | | |
| Yes | No | | Unclear | | Not applicable |  |

1. **JBI Prevalence studies checklist for** **Utuk and Atulomah (2023) (72)**

| Risk of Bias | | Response | | Justification | | |
| --- | --- | --- | --- | --- | --- | --- |
| Was the sample frame appropriate to address  the target population? | |  | | Study targeted Lafarge workers exposed to cement  dust. | | |
| Were study participants sampled in  an appropriate way? | |  | | Multistage selection described, but recruitment  procedures insufficiently detailed. Risk of  non-random workplace selection. | | |
| Was the sample size adequate? | |  | | Sample size calculation reported; n=238 justified. | | |
| Were the study subjects and the setting  described in detail? | |  | | Detailed demographic, workplace, and  environmental context provided. | | |
| Was the data analysis conducted with  sufficient coverage of the identified sample? | |  | | No response rate reported. Unclear how many  declined. Potential non-response bias. | | |
| Were valid methods used for the  identification of the condition? | |  | | Outcomes (knowledge, attitude, perception, HSB)  self-reported using self-constructed items. No  external validation. | | |
| Was the condition measured in a  standard, reliable way for all participants? | |  | | Questionnaire piloted and Cronbach’s α = 0.77  reported. Consistent scoring used. | | |
| Was there appropriate statistical analysis? | |  | | Descriptive statistics used appropriately.  Threshold classifications reported. | | |
| Was the response rate adequate, and if not,  was the low response rate  managed appropriately? | |  | | Response rate not reported. No non-response  follow-up, survey administered only to those present. | | |
| Yes | No | | Unclear | | Not applicable |  |
